# Abnormal Transcytosis Mechanisms in the Pathogenesis of Hydrocephalus: A Review

**DOI:** 10.3390/ijms26104881

**Published:** 2025-05-19

**Authors:** Adithi Randeni, Sydney Colvin, Satish Krishnamurthy

**Affiliations:** 1 Leeds General Infirmary, Great George Street, Leeds LS1 3EX, UK; adithi.randeni@nhs.net; 2 School of Osteopathic Medicine, Campbell University, 4350 US Hwy 421 S, Lillington, NC 27546, USA; scolvin507@gmail.com; 3 Neurosurgery Department, SUNY Upstate Medical University, Syracuse, NY 13210, USA

**Keywords:** hydrocephalus, transcytosis, macromolecular transport, efflux transporters, SNARE proteins, vesicle trafficking, membrane fusion, osmolarity gradients, hyh mice, pathogenesis of hydrocephalus

## Abstract

Hydrocephalus is a chronic neurological condition caused by abnormal cerebrospinal fluid (CSF) accumulation, significantly impacting patients’ quality of life. Its causes remain poorly understood, making neurosurgery the primary treatment. Research suggests that hydrocephalus may result from impaired macromolecular clearance, leading to increased osmotic load in the ventricles. Macromolecules are cleared via processes such as transcytosis, involving caveolae- and clathrin-dependent pathways, soluble N-ethylmaleimide-sensitive factor activating protein receptor (SNARE) proteins, and vesicular trafficking. Abnormalities in transcytosis components, such as mutations in alpha-SNAP (α-soluble NSF attachment protein) and SNARE complexes, disrupt membrane organization and vesicle fusion, potentially contributing to hydrocephalus. Other factors, including alpha-synuclein and Rab proteins, may also play roles in vesicle dynamics. Insights from animal models, such as hyh (hydrocephalus with hop gait) mice, highlight the pathological consequences of these disruptions. Understanding transcytosis abnormalities in hydrocephalus could lead to novel therapeutic strategies aimed at enhancing macromolecular clearance, reducing ventricular fluid buildup, and improving patient outcomes.

## 1. Introduction

### 1.1. Hydrocephalus

Hydrocephalus is a chronic neurological condition caused by an abnormal collection of cerebrospinal fluid (CSF) [1]. Hydrocephalus displays a bimodal age distribution, being most common in infancy and amongst the elderly population [2]. The global prevalence of hydrocephalus is about 85:100,000. However, in children, the prevalence is 88:100,000, with infantile hydrocephalus being between 1 and 32 per 10,000 births [2]. In all adults, the prevalence is about 11:100,000, but when the sample is limited to the elderly population, this increases to 175:100,000, and when looking at only those older than 80 years of age, the prevalence is 400:100,000 [2]. It has been found that hydrocephalus is more prevalent in the African and South American populations [2]. Very little is known about the causes of hydrocephalus despite the significant impact it has on the quality of life of patients [1,3,4]. As a consequence of this, patients with hydrocephalus have limited treatment options: they will need neurosurgery either for a shunt insertion or to undergo an Endoscopic Third Ventriculostomy (ETV) [5].

The pathophysiology of hydrocephalus is incompletely understood. The brain is a water-permeable organ [6], and several papers demonstrate that osmotic gradients have a role in the genesis of hydrocephalus [7,8]. In both acute and chronic rat models, introducing macromolecules into the ventricles has been shown to induce dose-dependent hydrocephalus by elevating the osmotic load [9,10]. Therefore, it is logical that macromolecular clearance from the ventricles would be a mechanism to establish the normal cerebrospinal fluid (CSF) osmolarity and consequently ventricular volume. Recent findings suggest that macromolecules are eliminated from the ventricles paravascularly via transcellular transport across the blood–brain barrier (BBB). They are eventually excreted in the urine. This elimination pathway has been confirmed in both healthy and hydrocephalic models [9]. However, the clearance of these macromolecules is notably delayed in the presence of hydrocephalus.

These data suggest that hydrocephalus results from a deficient clearance of macromolecules from the ventricles. Therefore, it is imperative to determine key mechanisms involved in the impairment of macromolecular clearance from the ventricles, which results in hydrocephalus. Our specific goal is to identify targets that modify macromolecular clearance and develop effective pharmacological interventions to relieve hydrocephalus. PubMed was searched using the keywords “Transcytosis” and “Hydrocephalus”, and the articles that were returned were reviewed for the purpose of this article.

### 1.2. Transcytosis

Transcytosis is one of three methods involved in molecular exchange in capillaries [11].

**Figure 1:**
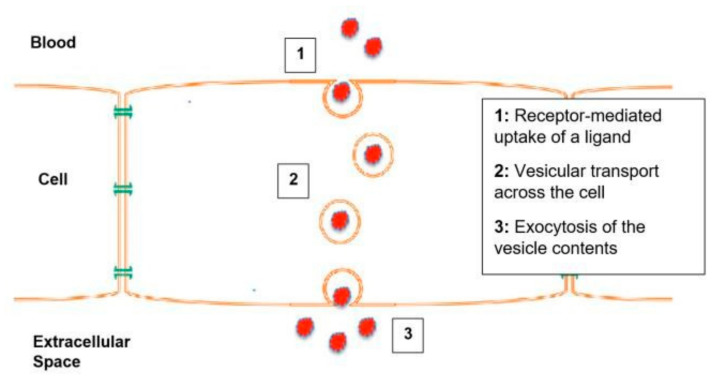
Simplified Diagram of Transcytosis [1,13].

**Figure 2:**
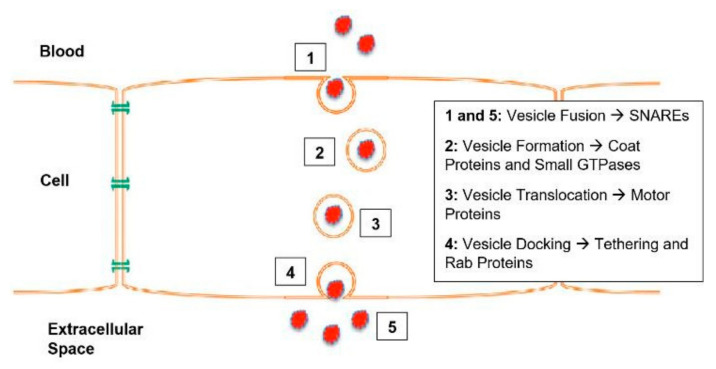
Different mediating factors of each step in transcytosis [13,14].

Transcytosis allows multicellular organisms to selectively transport molecules between distinct environments while maintaining the composition of each [12]. Transcytosis of macromolecules involves five steps: Equilibration of dissolved macromolecules in the capillary lumen with the fluid phase inside the open vesicle; pinching off of the vesicle; vesicle shuttling to the cytoplasm and transient fusion with other vesicles within the cytoplasm, allowing intermixing of the vesicular content; fusion of vesicles with the opposite plasma membrane; and equilibration with the opposite extracellular fluid phase [1]. A simplified diagram of transcytosis is shown in Figure 1, and Figure 2 shows the different factors that mediate each step in the transcytosis process.

Transcytosis can be further divided into caveolae-mediated transcytosis and clathrin-mediated transcytosis [11]. Table 1 summarises the differences between the two types.

Regardless of the type of transcytosis employed, intercompartmental transport and exocytosis depend on membrane fusion [15]. Thus, it is necessary to understand the role Soluble N-ethylmaleimide-sensitive factor activating protein receptor (SNARE) complexes play in membrane trafficking, as they are the core machinery mediating membrane fusion [14]. SNARE function and complex assembly are thought to integrate multiple regulatory pathways that coordinate membrane trafficking [14].

### 1.3. SNARE Complexes and the SNARE Hypothesis

SNARE proteins belong to a large superfamily of small, membrane-anchored proteins thought of as engines for membrane fusion [16]. Over 38 SNARE proteins have been characterized to mediate specific transport events [17].

SNARE proteins are composed of a conserved 60–70 amino acid SNARE motif arranged in heptad repeats [16]. Although unstructured in isolation, these motifs assemble into a four-helix bundle forming the SNARE core complex, with each helix from a distinct SNARE subfamily [16]. Most SNAREs are membrane-anchored via C-terminal transmembrane domains, while some are attached through lipid modifications, including palmitoylation or prenylation [14].

**Figure 3:**
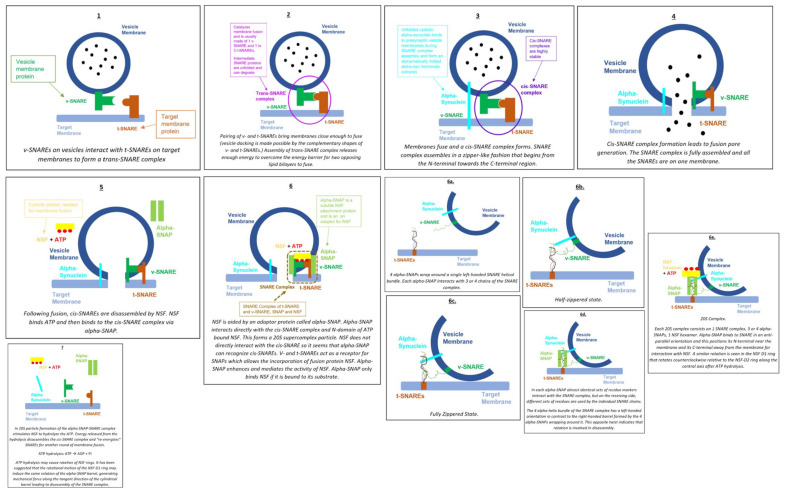
The SNARE Hypothesis [14,15,16,17,18,19,20,21,22,23,24,25,26,27,28].

Figure 3 below describes the current SNARE Hypothesis and the factors that have been thought to mediate it. This paper will look at these various components involved in transcytosis of macromolecules, particularly those involved in membrane fusion, and explore their links to the formation of hydrocephalus.

## 2. N-Ethylmaleimide Sensitive Factor (NSF)

### 2.1. Introduction

N-ethylmaleimide Sensitive Factor (NSF), a type II AAA+ hexameric ATPase, was one of the initial proteins of the cell trafficking mechanism to be identified [12]. All AAA+ proteins transduce ATP hydrolysis into major conformational changes that remodel client proteins [16]. NSF plays a crucial role in membrane trafficking and serves as a key component in SNARE-mediated fusion and events, including neurotransmitter release [29,30]. Soluble NSF attachment protein (SNAP) is a cofactor of NSF, and together the two specialize in disassembling highly stable SNAP receptor protein (SNARE) complexes to promote intramembrane cellular trafficking [16,31].

### 2.2. Mechanism

As shown in Figure 3, NSF disassembles the cis-SNARE complex [16]. Similar to other AAA+ proteins, NSF monomers assemble to form a homohexamer [16]. Each monomer consists of the N-terminal domain and two AAA+ domains [16]. The overall positive charge of the N-terminal domains is thought to interact with the C-terminal negative charge distribution of alpha-SNAP [16]. The N-terminal of NSF is also thought to interact with the SNARE complexes, but there is little evidence to support this [16]. It should be noted that NSF only binds to SNAP in the presence of ATP [16,18]. Wang et al. discovered that SNAP binds to the SNARE complex with an opposing structural twist and that the conversion of NSF from its ATP-bound to adenosine diphosphate-bound (ADP-bound) form drives conformational changes, which ultimately facilitate SNARE complex disassembly [14].

In eukaryotes, there are many different sets of SNARE complexes required for different steps in transcytosis [16]. However, NSF and SNAP are universal and can recognize and disassemble nearly all characterized SNARE complexes [16]. An exception occurs when SNARE complexes are shielded by binding partners (including SM-Sec1/Munc18-like proteins), which prevents disassembly by NSF and SNAP [16].

NSF and SNAP also seem to have a proof-reading capability in the assembly of SNARE complexes, as premature and incorrect complexes are disassembled in their trans-conformation [16].

### 2.3. NSF and Hydrocephalus

When mutations in NSF in relation to hydrocephalus were searched using PubMed, several articles were obtained in which a mutation in alpha-SNAP was correlated to hydrocephalus in hyh mice (hydrocephalus with hop gait) [29,30,32,33,34,35,36,37,38,39,40]. Seeing as alpha-SNAP interacts with NSF during exocytosis, this is an important link in establishing the transcytosis of macromolecules via this pathway [18]. The alpha-SNAP mutations in relation to hydrocephalus will be dissected in the alpha-SNAP section, as the mutations discussed are associated with alpha-SNAP instead of NSF.

## 3. Alpha-SNAP

### 3.1. Introduction

Alpha-SNAP (alpha-soluble NSF attachment protein) is coded by the N-ethylmaleimide-sensitive factor Attachment Protein Alpha (NAPA) gene on Chromosome 7 and is a ubiquitous protein that is required for membrane fusion in eukaryotes [19,33,41]. It is a member of the SNAP family and has been found to be conserved between several species, indicating its importance [41]. Mammals express three SNAP isoforms, including α-, β-, and γ-SNAP, respectively, with α-SNAP being the most prevalent and widely distributed form [41]. NSF and alpha-SNAP only disassemble cis-SNAREs and partially misassembled SNARE complexes, not trans-SNAREs [19]. In the cytoplasm, alpha-SNAP binds to SNAREs in a 1:1 ratio, but on the membranes or with NSF, it binds to SNARE complexes anywhere from a 1:1 ratio to a 1:4 ratio [19].

### 3.2. Mechanism

As seen in Figure 3, alpha-SNAP works alongside NSF to disassemble SNARE complexes [16]. SNARE complexes contain a parallel four-helix bundle, a two-stranded coiled coil bridging the linker and transmembrane domain, and an N-terminal regulatory domain (NRD) [19]. The NRD comprises two functionally distinct regions: a short N-terminal peptide of roughly 15 amino acids and a self-folding three-helix bundle known as the Habc domain [19]. The structure of the alpha-SNAP-SNARE subcomplex was illustrated in a paper by Huang et al. in 2019 [20]. R116A and L197A residues were suggested to have direct roles in SNARE complex disassembly, as mutations in these residues had an average effect on the binding activity [20]. A significant portion of alpha-SNAP is believed to mediate its interaction with the SNARE complex [15]. Evidence from shape complementarity, sequence conservation, and surface charge distribution suggests that the concave face or extended edge of alpha-SNAP’s twisted sheet domain may be responsible for binding the SNARE complex [15].

### 3.3. Alpha-SNAP and Hydrocephalus

Deletion of alpha-SNAP in mice has been found to be lethal, and Chae et al. found that mutation of alpha-SNAP caused hydrocephalus in mice [35].

In 2006, a paper described a study conducted on 3017 mice, 22.4% hydrocephalic, to determine how the mutations in hyh mice lead to hydrocephalus [33]. Mutant hyh mice develop an autosomal recessive inherited hydrocephalus [33]. Hyh mice carry a missense mutation in the Napa gene, leading to the substitution of methionine for isoleucine at position 105 (M105I) of alpha-SNAP [41]. The M105I mutant protein has been found to be 70% less abundant than the normal wild-type protein [39].

In hyh mice, the primary defect occurs in the ependymal cells, which detach and undergo programmed, progressive denudation of the ependymal lining along the ventral walls of the ventricles and the Sylvius aqueduct [36]. Postnatally, this process extends to the dorsal ependyma of the Sylvius aqueduct, thus resulting in ventral and dorsal neuropile fusion, aqueduct obstruction, and severe hydrocephalus [36]. The mutated alpha-SNAP in hyh mice results in altered localization of surface proteins in the neuroepithelial cells and neural stem cells [33]. It is possible that this defect would also be expressed in the ependymal cell lineage and cause ependymal detachment in the hydrocephalic mice [33].

A study by de Paola et al. reported that hyh mice exhibit structural and behavioral abnormalities in addition to miscolocalization of apical proteins in neuroepithelial cells, resulting from a homozygous point mutation (Met105Ile; M105I) in alpha-SNAP [40]. Although this single amino acid substitution does not disrupt alpha-SNAPs binding to NSF or its role in NSF-mediated SNAE complex disassembly, the mutation has been hypothesized to affect cell polarity through altering liver kinase B1 (LKB1)-AMPK (Adenosine Monophosphate Activated Protein Kinase) signalling [40]. Both LKB1 (liver kinase B1) and AMPK (AMP-activated protein kinase) are critical regulators of cell polarity. Data from Wang and colleagues suggest that the M105I mutation confers a gain-of-function, increasing alpha-SNAP’s affinity for AMPK and subsequently dampening LKB1-AMPK pathway activity [40]. However, the precise mechanism by which reduced AMPK activity contributes to the hyh phenotype remains unclear.

Other papers have also found evidence that suggests that the mutation in hyh mice alpha-SNAP affects apical protein localization in neuroepithelial cells but does not affect NSF function or SNARE disassembly [30,34,35,39]. Impaired alpha-SNAP-mediated membrane trafficking has been proposed to disrupt cell–cell adhesion, potentially leading to membrane disorganization and neuronal ectopia [42]. It is possible that the mutation, in a similar manner, affects transmembrane transport in the brain by affecting cell polarity, thus leading to hydrocephalus.

Work by Miao et al. implies that the mutations in alpha-SNAP may cause hydrocephalus by disrupting sodium influx [36]. The paper focused on Cluster of Differentiation 4 (CD4) T cells and Orai1 channels. However, the underlying principle is worth discussing. Alpha-SNAP is required in the functional assembly of Orai1 multimers [36]. T cell effectors require calcium influx, and the paper looks at the non-specific permeation of sodium via the calcium channels [36]. In hyh mice, T cell receptor stimulation induced rapid sodium influx [36]. Ablation of the Orai1 corrected the disruptions caused by the rapid sodium influx [36]. This indicates that alpha-SNAP mutations may impact calcium channels (which in turn can impact non-specific sodium influx via these calcium channels) and lead to hydrocephalus.

Therefore, the main model for understanding hydrocephalus in the presence of alpha-SNAP mutations is the hyh mouse. In this model, the main mechanism for hydrocephalus formation is that the alpha-SNAP mutation leads to defects in membrane organisation and adhesion, which has a smaller impact on SNARE disassembly. It is not yet known if the reduced expression of the mutated protein impacts hydrocephalus formation in hyh mice. Further investigations are required to determine how the alpha-SNAP mutation in hyh mice leads to hydrocephalus.

## 4. Alpha-Synuclein

### 4.1. Introduction

**Figure 4:**
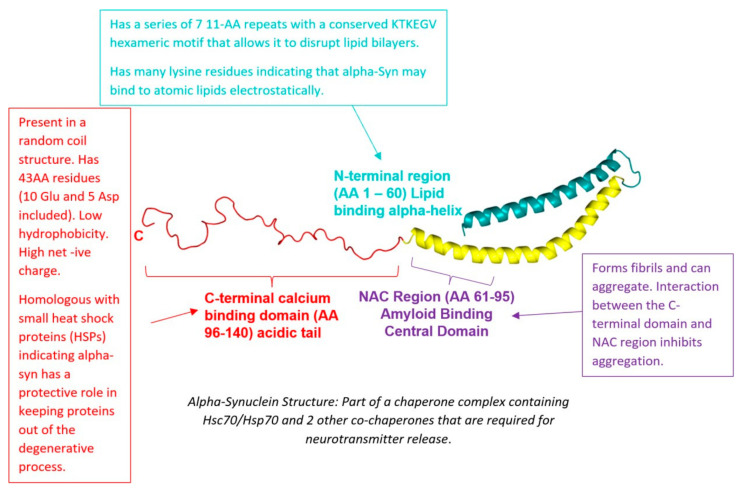
The Structure of Alpha-Synuclein [21,42,44].

Human alpha-synuclein (α-syn) is predominantly expressed in the brain and is encoded by the SNCA gene (also known as NACP or PARK1) located on chromosome 4q21. The protein is composed of 140 amino acids encoded by six exons [21,43]. Structurally, α-syn may adopt an α-helical conformation when bound to phospholipid membranes or remain unfolded in the cytosol. These conformational states suggest that α-syn may have distinct functional roles depending on its cellular localization and structure. The predominant form of α-syn is the full-length protein, but shorter isoforms exist [21]. The structure of alpha-synuclein is shown in Figure 4, and its functions are summarised in Table 2.

### 4.2. Mechanism

Table 2 shows that alpha-synuclein has multiple roles.

SNARE Complex Assembly: To allow presynaptic vesicles to release neurotransmitters multiple times, SNARE assembly and disassembly are required. This chaperone assembly of the SNARE complex leads to neuroprotection in presynaptic terminals. Conversely, direct interaction between α-syn and the SNARE protein synaptobrevin-2 (vesicle-associated membrane protein 2) inhibits the assembly of the SNARE complex. Further, large oligomers of α-syn binding to the N-terminal domain of synaptobrevin-2 SNARE protein prevent assembly of SNARE complexes and lead to neurodegeneration. Therefore, the interaction of α-syn with either the membrane or the SNARE complex allows the mediation of SNARE complex assembly and disassembly, and mutations in α-syn can lead to a deficiency in SNARE complex formation [21].

Molecular Chaperone: Heat shock proteins (HSPs) are molecular chaperones expressed in response to various cellular stresses. One such chaperone, cysteine string protein alpha (CSPα), is localized to the outer membrane of presynaptic vesicles and plays a critical role in neurotransmitter release. Experimental studies have shown that deletion of CSPα results in reduced α-syn levels and is associated with lethal neurodegeneration. The CSPα knockout mice can be rescued if human wild-type α-syn is overexpressed, confirming that α-syn can recover a chaperone activity [21]. Thus, mutations in α-syn may lead to disability of chaperone activity and thus reduce transmembrane trafficking activities such as neurotransmitter release.

Cellular and Intracellular Membranes: Oxidative stress in neurons can initiate pathological processes. The oxidation of unsaturated phospholipids can damage and disrupt both cellular and intracellular membranes. The disruption of lipid membranes in the brain could lead to unmediated movement of molecules between membranes, resulting in the formation of hydrocephalus. The monomeric form of α-syn can protect lipids from oxidation by interacting with lipid membranes. Through either mechanism (either as an antioxidant or by preventing apoptosis), α-syn can help keep cellular and intracellular membranes intact [21]. This could suggest a role for α-syn in preventing the formation of hydrocephalus by allowing cells to retain their ability to mediate membrane trafficking of molecules.

**Figure 5:**
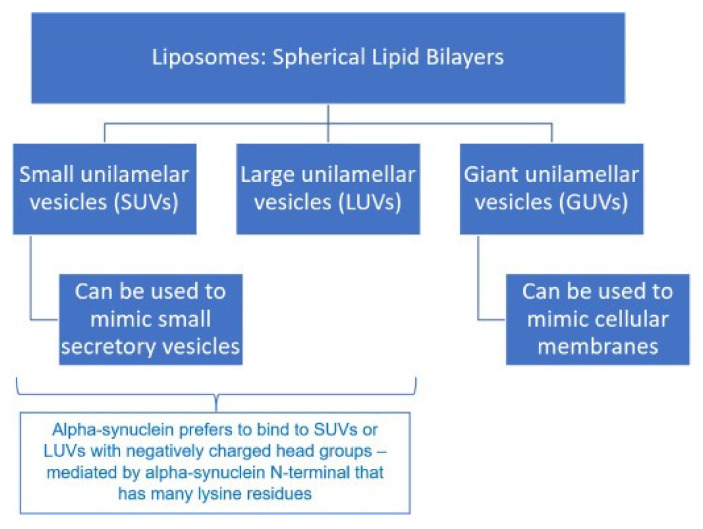
Types of Liposomes [21].

Vesicle Trafficking: α-syn’s ability to interact with lipid membranes is the main reason for its role in modulating vesicle trafficking. Liposomes are spherical lipid bilayers that are classified into three types, as shown in Figure 5.

**Figure 6:**
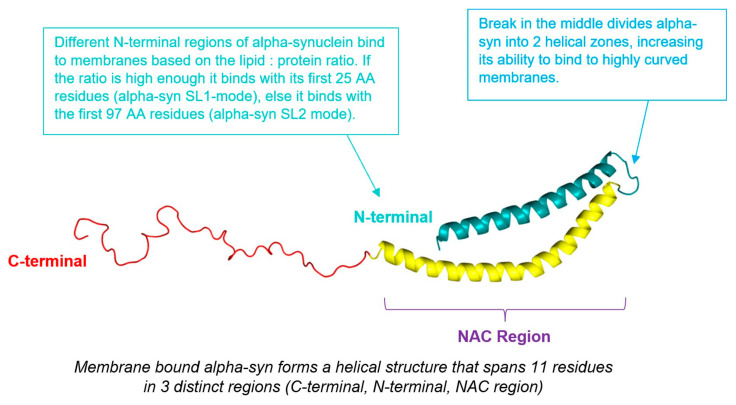
Membrane-Bound Alpha-Synuclein [21,44].

α-syn seems to prefer some acidic head groups over others. Polyunsaturated acyl chains enhance α-syn’s ability to associate with membranes, likely due to the increased spacing between loosely packed unsaturated lipids [21]. Further, α-syn takes on different attributes when bound to curved membranes, as illustrated by Figure 6.

Thus, α-syn binds selectively to the curved presynaptic membranes and modulates vesicle trafficking. α-syn can detect vesicle pools due to its high affinity for lipid bilayers and its ability to sense membrane curvature, making it particularly enriched in pools containing numerous small liposomes. It has been shown to reduce both the quantity and rate of synaptic vesicle recycling back to the presynaptic terminal. This reduction is important for synaptic homeostasis [21].

### 4.3. α-Synuclein and Hydrocephalus

α-syn interacting with membranes may also cause α-syn to aggregate. Aggregation of α-syn produces protofibrils, which create pore-like openings in membranes, thus resulting in vesicle content leakage. Deletion of residues 74–84 prevents this aggregation. This indicates that the central NAC region (capable of forming beta-sheet structures) is primarily responsible for the membrane disruption [21]. There is no research looking at whether α-syn-mediated membrane disruption causes hydrocephalus. However, it is reasonable to assume that the leakage of vesicular contents can disrupt membrane transport and lead to abnormal accumulations of macromolecules in the CSF spaces, which in turn could result in hydrocephalus.

The main link that has been established between α-syn, synucleopathies, and hydrocephalus is the presence of comorbid α-synucleopathies in idiopathic normal pressure hydrocephalus (iNPH) [43,45,46,47,48]. A study that included 79 patients with iNPH found that 23 patients also developed comorbid Parkinson’s Disease or Parkinson’s Disease Dementia, and eight patients developed comorbid Dementia with Lewy Bodies [45]. In iNPH, irregular pressure waves, and not physical obstruction, cause the altered CSF fluid dynamics [47]. In a study with 39 patients, posterior cingulate cortex magnetic resonance imaging (PCC MRI) found that in patients with iNPH, there was hyperdynamic CSF flow at the level of the cerebral aqueduct [47].

## 5. t-SNAREs and v-SNAREs

### 5.1. Introduction

**Figure 7:**
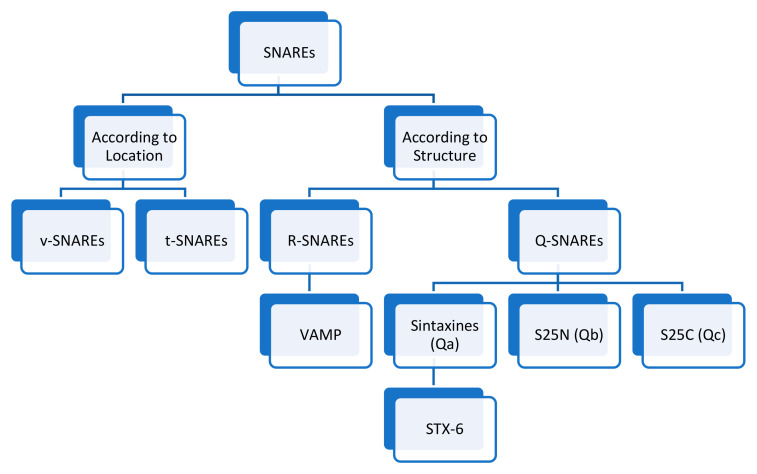
Different types of SNAREs [23].

As discussed previously, there are different types of v- and t-SNAREs (Figure 7). The differences are summarised in Table 3 [23].

### 5.2. Mechanism

**Figure 8:**
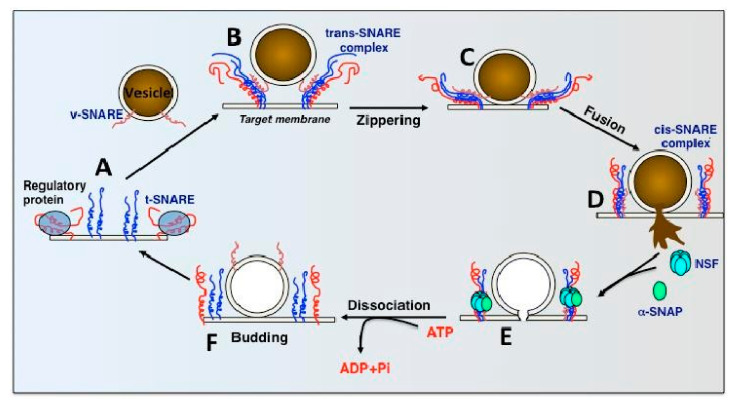
Vesicle Fusion mediated by SNAREs [24,25].

The Rothman proposal (also known as the SNARE Hypothesis, shown in Figure 3), as discussed in the introduction of this paper, hypothesizes a universal “docking and fusion” particle to explain vesicle docking and fusion at all locations [24]. The process of vesicle fusion mediated by SNAREs is shown in Figure 8.

### 5.3. v-SNAREs/t-SNAREs and Hydrocephalus

Membrane trafficking is critical for cellular homeostasis and numerous physiological processes. Therefore, complete loss of function in NSF, SNAPs, or most SNARE proteins results in cell death and embryonic lethality [49]. However, point mutations can be employed to determine the roles and functions of the different components of cellular trafficking [50].

A study by Nechiporuk et al. showed that discs large homolog 5 (Dgl5) (−/−) mice develop fully penetrant hydrocephalus and kidney cysts [51]. Dig5 encodes an evolutionarily conserved coiled-coil membrane-associated guanylate kinase (MAGUK) protein essential for maintaining epithelial tube integrity in the brain and kidneys [52]. The development of these phenotypes is thought to result from impaired membrane delivery of cadherin–catenin adhesion complexes and subsequent loss of cell polarity [52]. Dig5 has been shown to travel with cadherin-containing vesicles and interact with the t-SNARE protein syntaxin 4 [52]. Nechiporuk and colleagues propose that Dig5 facilitates the targeted delivery of cadherins to the plasma by linking cadherin-loaded vesicles to the t-SNARE fusion machinery [52]. This highlights the critical role of cadherin trafficking in maintaining cell polarity and epithelial tube architecture, processes vital for normal brain development [52].

## 6. Munc18 (Sec 1) Protein

### 6.1. Introduction

Earlier in this paper, the possibility that alpha-SNAP recognizes cis-SNAREs and thus only allows NSF to disassemble cis-SNAREs and not trans-SNAREs was mentioned. However, it has also been suggested that proteins such as mammalian uncoordinated-18 (Munc18) protect trans-SNAREs from NSF and alpha-SNAP disassembly [19]. Studies in mice deficient in munc18a have shown that the protein is also necessary for exocytosis in mammals [26].

### 6.2. Mechanism

**Figure 9:**
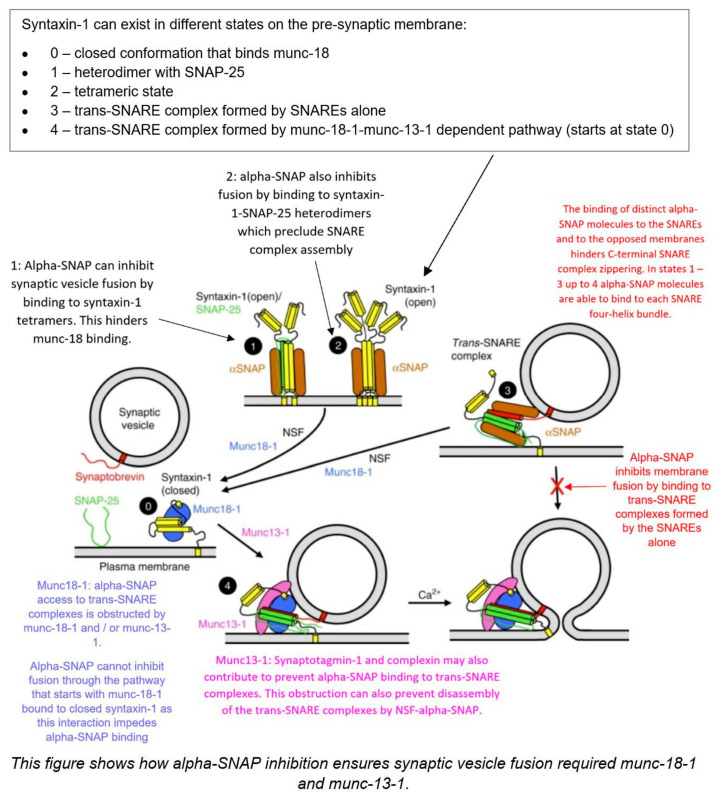
Role of Munc Proteins in Exocytosis [27].

Munc18-1 and munc13-1 organize SNARE complex formation by an NSF-SNAP-resistant mechanism [27]. Figure 9 illustrates the importance of munc-18-1 and munc-13-1.

### 6.3. Munc18 (Sec1) Protein and Hydrocephalus

Mutations to munc18-1 can modulate the stability of the template complex, which shows that chaperoned SNARE assembly is essential for exocytosis [53]. Knockout or mutagenesis of munc18 in various organisms results in significantly decreased secretion, highlighting its essential role in promoting exocytosis [49].

## 7. Rab Proteins

### 7.1. Introduction

**Figure 10:**
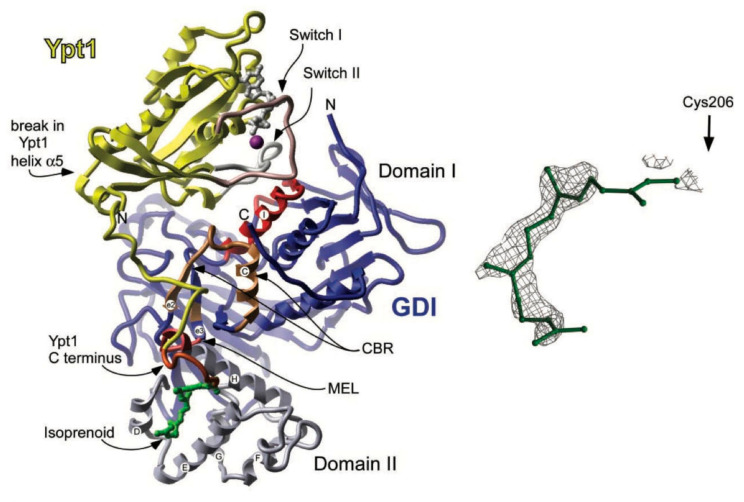
Generalised Structure of Rab Proteins [28,52]. From [52], reprinted with permission from AAAS.

Figure 10 illustrates the generalized structure of Rab proteins. Table 4 summarizes the main functions of Rab Proteins.

### 7.2. Mechanism

**Figure 11:**
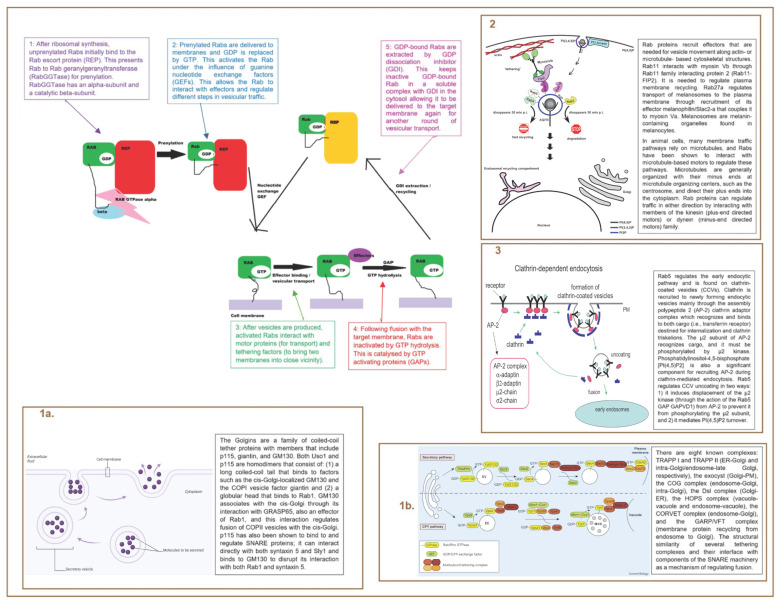
Cycling of Rab Proteins and Functions of Rab Proteins—Molecular Switch; Cargo Selection and Vesicle Formation; Vesicle Movement; Vesicle Uncoating; Vesicle Tethering; and Membrane Fusion [17,28,54,55,56,57,58].

Molecular Switches: Rab proteins continuously cycle between the cytosol and different membranes [54]. Rab proteins have a multitude of other functions as well [17,54]. These are illustrated in Figure 11 [17,28,54] below.

Cargo Selection and Vesicle Formation: The cargo in vesicles is selected by components of the coat complex of each vesicle [28]. These proteins recognize specific cargo elements targeted for transport [28]. Rab proteins facilitate this process by recruiting coat complexes and additional effectors necessary for vesicle formation [28].

Vesicle Tethering: To ensure accurate membrane transport, most pathways require factors that “tether” vesicles to their target membrane prior to fusion. These tethering factors are generally classified into two types: long coiled-coil proteins or multiprotein complexes [28]. Both types function as Rab effectors, with some also modulating the nucleotide-binding state for their associated Rab proteins [28]. Collectively, these tethers play a critical role in regulating SNARE-mediated vesicle fusion with the target membrane [28].

Membrane Fusion: Rab proteins regulate SNARE-dependent membrane fusion between transport vesicles and their target membranes [28]. They can interact directly with SNARE proteins or indirectly through SNARE regulatory proteins such as SM or lethal giant larvae (Lgl) [28].

### 7.3. Rab Proteins and Hydrocephalus

Rab proteins are essential for neuronal function [59]. The Rab GTPase family is known to be related to many diseases [28]. Research has found that RabL2A is essential for the normal function of cilia and flagella [60]. Mutations in this gene lead to ciliopathies [60]. While there is no direct link that has been established between this ciliopathy and hydrocephalus, a possibility is that the same mechanisms leading to ciliopathy impair membrane vesicular transport, thereby contributing to hydrocephalus formation.

Cilia are hair-like organelles that extend from the surface of eukaryotic cells and play crucial roles in motility, sensory reception, and intracellular transport. These structures are classified into motile and non-motile (primary) cilia, each serving distinct functions. Motile cilia generate coordinated, ATP-dependent beating patterns that drive fluid movement across epithelial surfaces, such as in the respiratory tract and fallopian tubes. The movement of cilia is powered by dynein, an ATPase motor protein that facilitates the sliding of microtubule doublets within the axoneme, the structural core of cilia. This activity generates the characteristic wave-like motion necessary for fluid transport and cellular signalling [61].

Beyond their role in motility, cilia are closely linked to vesicular transport processes. Intraflagellar transport (IFT) is a key mechanism by which cilia are maintained and functionally regulated. IFT relies on ATP-driven motor proteins—kinesin-2 for anterograde transport and cytoplasmic dynein-2 for retrograde transport—to shuttle protein cargo along the microtubule tracks within the cilium [62]. This transport system is essential for delivering membrane proteins, receptors, and signalling molecules required for ciliary function. Moreover, vesicular transport and ciliary function are interconnected in cellular homeostasis and signalling. For example, exocytosis and endocytosis at the ciliary base regulate the availability of signalling receptors, such as those involved in the Hedgehog pathway [63]. Dysregulation in ATP-dependent ciliary and vesicular transport mechanisms has been implicated in ciliopathies—disorders characterized by defects in cilia structure or function, leading to diseases such as polycystic kidney disease and Bardet–Biedl syndrome.

Overall, ATP-driven ciliary movement and vesicular transport are intricately linked processes that facilitate cellular communication, signalling, and material exchange. By coordinating vesicle trafficking with ciliary function, cells ensure the precise localization of receptors and signalling molecules necessary for normal physiological processes. Further studies on these ATP-dependent mechanisms may provide insights into potential therapeutic strategies for ciliopathies and other transport-related disorders.

Impaired L1 cell adhesion molecule (L1CAM) function has been found to underlie X-linked L1 syndrome, which encompasses congenital hydrocephalus [64]. Previous studies have identified a genetic interaction between Caenorhabditis elegans L1CAM, encoded by the sax-*7* gene, and RAB-3 [65]. Therefore, there may be an indirect link between congenital hydrocephalus caused by impaired L1CAM function and Rab protein dysfunction.

Griscelli syndrome is a rare, autosomal recessive disorder [65]. Primary neurological presentation is rare in this disease, and there has been a case that presented with obstructive hydrocephalus [65]. This case also had a RAB 27A mutation [65]. While the link between Griscelli syndrome and hydrocephalus has not been established, it is possible that the RAB 27A mutation played a role in the formation of either Griscelli syndrome or hydrocephalus or both. Another study identified a RAB23 mutation in the homozygous state in four related individuals of Comorian origin diagnosed with Carpenter syndrome [65]. Brain imaging showed hydrocephalus in two out of four children [65]. No link has been established between RAB23 and hydrocephalus formation.

Table 5 summarises the key information discussed in the body of this article.

## 8. Conclusions

The process of transcellular transport is complex and has many proteins and factors that regulate it. It has been shown that the pathogenesis of hydrocephalus is a result of poor macromolecular clearance out of the ventricles. This review reveals that abnormal transcytosis can result in hydrocephalus. Therefore, transcytosis could be one of the mechanisms that help clear these macromolecules. However, very little research has been performed on the link between transcytosis and hydrocephalus formation. Thus, further work in this area can help shed light on the mechanisms behind the formation of hydrocephalus and lead to a better understanding of the disease. It can also lead to better management strategies for hydrocephalus, particularly possible pharmacological strategies focusing on improving or promoting transcytosis mechanisms to improve the clearance of macromolecules.

## Figures and Tables

**Table 1:** Differences between caveolae-mediated and clathrin-mediated transcytosis [11].

Caveolae Mediated	Clathrin Mediated
Make us of caveolae	Make use of clathrin protein
Caveolae are distinguished by the presence of caveolins and are pits in the apical and basal membranes of all endothelial cells	Clathrin is located on both apical and basal surfaces of epithelial cells and line these vesicles. On the surfcae of the cell a pit forms from specific cell receptors that are coated by clathrin
Mostly responsible for transcellular translocation of macromolecules in epithelal cells	Used by epithelial cells to sort through the molecules entering the cell as one of the destinations of these vesicles is the Golgi. Vesciles attach to the endoplasmic reticulum before being sorted to either the apical or basal side of the cell
Transport cargi, usually fluid, through the cells	Required for immune responses
Caveolae can merge to create arrangements including a tunnel or channel, to move cargo through the cell	Clathrin stabilizes the forming vescile by forming a rigid matrix of an assembling of clathrin proteins, which can later disassemble after the vesicle has disassociated from the membrane

**Table 2:** Roles of Alpha-Synuclein [42].

Molecules chaperone and assists in the folding of SNAREs at pre-synaptic plasma membranes alongside cysteine string protein-alphs/DNAJC5 Chaperone activity is essential in maintaining normal SNARE complex assembly during aging
Regulate dopamine neurotransmission and modulates its activity via the dopamine transporter (DAT1)
Neuronal protein that regulates synaptic vesicle trafficing and neurotransmitter release
Promotes vesicle priming, fusion and dilation of exocytotic fusion pores
Increases local Ca^2+^ release from microdomains Required for the enhancement of ATP-induced exocytosis

**Table 3:** Differences between v-SNAREs and t-SNAREs [23].

v-SNARES	t-SNARES
Found on membrane transport vesicle during the budding process of exocytosis (usually incorporated into the membrane of transport vesicles)	Associated with nerve terminal membranes
e.g., VAMP7 and VAMP8	e.g., Syntaxin 1 and SNAP-25
Have more than 70% of branced amino acids in the transmembrane region	Form stable subcomplexes and function as a guide for v-SNAREs
Aid exocytosis of large zymogen granules and mast cell vesicles	Specific localization to subcellular membranes defines where transport vesicles bind and fuse
Facilitate rapid pore expansion and release of bulky macromolecules such as interferons	

**Table 4:** Functions of Rab Proteins [28].

Regulate protein transport in endocytic and exocytic pathways
Participate in vesicle budding, membrane fusion and cytoskeletal interactions
Act as key regulators in intracellular vesicular transport
Regulate pathways by interacting with effector proteins: Effector proteins are proteins that preferentially interact with the GTP-bound forms of the Rab Different Rab effectors act during vesicle formation, movement, tethering and fusion

**Table 5:** Table summarising the key points discussed within this article.

	Introduction	Mechanism	Link to Hydrocephalus
NSF	NSF is a type II AAA+ hexameric ATPase involved in cell trafficking. It is vital for SNARE-mediated membrane fusion, including neurotransmitter release. NSF works with its cofactor SNAP to disassemble stable SNARE complexes and facilitate intracellular transport.	NSF forms a homohexamer composed of an N-terminal and two AAA+ domains. The N-terminal domain interacts electrostatically with alpha-SNAP. NSF requires ATP to bind SNAP and disassemble SNARE complexes. SNAP binds SNARE complexes with an opposing twist; ATP hydrolysis by NSF drives disassembly. NSF and SNAP are broadly effective across SNARE complexes, except when shielded by proteins like SM. They also correct misassembled SNARE complexes.	No direct mutations in NSF linked to hydrocephalus were found. Mutations in alpha-SNAP are linked to hydrocephalus in hyh mice. Since alpha-SNAP interacts with NSF, this suggests a potential pathway affecting macromolecular transcytosis.
Alpha-SNAP	Alpha-SNAP is encoded by the *NAPA* gene on Chromosome 7 and is crucial for membrane fusion in eukaryotes. It is the most prevalent of the three SNAP isoforms (α, β, γ) and is conserved across species. Disassembles cis-SNARE and misassembled complexes (not trans-SNAREs). Binds SNAREs at a 1:1 ratio in the cytoplasm; varies (1:1–1:4) on membranes or with NSF.	Works with NSF to disassemble SNARE complexes. SNAREs include a four-helix bundle and an N-terminal regulatory domain (NRD). Mutations in alpha-SNAP residues (R116A, L197A) affect SNARE binding. Likely binds SNARE via the concave face or the extended edge of its twisted sheet domain.	Deletion of alpha-SNAP is lethal; mutations linked to hydrocephalus in *hyh* mice. *hyh* mice carry a missense mutation (M105I) in *NAPA*, reducing alpha-SNAP abundance by ~70%. Causes ependymal cell detachment and aqueductal obstruction, leading to severe hydrocephalus. Mutation mislocalises apical proteins in neuroepithelial cells. Homozygous point mutation (Met105Ile; M105I) in alpha-SNAP is suggested to affect cell polarity via disrupted LKB1-AMPK signalling, possibly a gain-of-function mutation. Impacts membrane trafficking, possibly leading to membrane disorganization and neuronal ectopia. May disrupt sodium influx through Orai1 channels, affecting calcium homeostasis. The main pathological model is the *hyh* mouse. Alpha-SNAP mutation led to defects in membrane organisation and adhesion and had a smaller impact on SNARE disassembly, but more research is needed to confirm mechanisms.
Alpha-Synuclein	α-syn is encoded by the *SNCA* gene on chromosome 4q21. Composed of 140 amino acids across six exons. Adopts α-helical conformation when membrane-bound or remains unfolded in the cytosol. Multiple isoforms exist, but full-length α-syn is predominant.	SNARE Complex Assembly: Facilitates SNARE complex assembly for neurotransmitter release, offering neuroprotection. Direct binding to synaptobrevin-2 can inhibit SNARE assembly. Large oligomers block SNARE function and contribute to neurodegeneration. Mutations can impair SNARE formation. Molecular Chaperone: α-syn may compensate for CSPα chaperone loss. CSPα knockout mice show lethal neurodegeneration, rescued by α-syn overexpression. Suggests α-syn mutations may impair chaperone function and neurotransmitter release. Cellular and Intracellular Membranes: α-syn protects neuronal membranes from oxidative damage. Prevents apoptosis and helps maintain membrane integrity. May prevent hydrocephalus by preserving intracellular transport systems. Vesicle Trafficking: Binds selectively to curved presynaptic membranes due to affinity for lipid curvature. Interacts with polyunsaturated lipids. Modulates vesicle trafficking and reduces synaptic vesicle recycling for synaptic homeostasis.	Aggregation of α-syn creates protofibrils that disrupt membranes and cause vesicle content leakage. The central NAC region is responsible for aggregation (residues 74–84). Potential link between α-syn-mediated membrane disruption and hydrocephalus via macromolecule buildup in CSF. α-syn pathology observed in idiopathic normal pressure hydrocephalus (iNPH). Studies show the co-occurrence of Parkinson’s Disease and Dementia with Lewy Bodies in iNPH patients. In iNPH, irregular pressure waves, and not physical obstruction, cause the altered CSF fluid dynamics.
t-SNARES and v-SNARES	There are various types of v-SNAREs and t-SNAREs involved in membrane trafficking (see Figure 7). Rothman’s SNARE Hypothesis proposes a universal “docking and fusion” mechanism for vesicle transport (see Figure 3 and Figure 8).	Membrane trafficking is essential for cellular function and organismal development. Complete loss of NSF, SNAPs, or SNARE proteins results in embryonic lethality. Point mutations help study specific SNARE roles.	Dlg5 knockout (Dlg5-/-) mice develop hydrocephalus and kidney cysts. Dlg5 encodes a membrane-associated guanylate kinase (MAGUK) protein that maintains epithelial tube integrity. Dlg5 interacts with t-SNARE syntaxin 4. Dlg5 travels with cadherin-containing vesicles and links them to SNARE machinery. Loss of Dlg5 impairs cadherin delivery, disrupting cell polarity and leading to hydrocephalus. Dig5 facilitates the targeted delivery of cadherins to the plasma by linking cadherin-loaded vesicles to the t-SNARE fusion machinery. Highlights the importance of SNARE-linked polarity.
Mun18 (Sec1) Proteins	Alpha-SNAP allows NSF to disassemble cis-SNAREs, not trans-SNAREs. Munc18 may protect trans-SNAREs from NSF/alpha-SNAP disassembly. Munc18a is essential for exocytosis in mammals.	Munc18-1 and Munc13-1 coordinate SNARE complex formation. This pathway is resistant to NSF/alpha-SNAP, enabling proper membrane fusion. See Figure 9 for a detailed illustration.	Mutations in munc18-1 affect template complex stability, emphasizing its role as a chaperone in SNARE assembly. Knockout/mutagenesis of munc18 leads to reduced secretion, demonstrating its critical role in exocytosis.
Rab Proteins	Rab proteins have a conserved structure (see Figure 10). Their functions are summarized in Table 4.	Molecular Switches: Cycle between the cytosol and membranes. Function in vesicle trafficking, fusion, and signalling. Cargo Selection and Vesicle Formation: Recruit coat proteins and effectors for vesicle formation. Vesicle Tethering: Ensure accurate docking before membrane fusion. Use long coiled-coil proteins or multiprotein complexes. Membrane Fusion: Regulate SNARE-mediated fusion via direct/indirect SNARE interactions. Indirectly through SNARE regulatory proteins such as SM or lethal giant larvae (Lgl)	RabL2A mutations disrupt cilia/flagella function → potential link to hydrocephalus via impaired vesicular transport. Cilia function is ATP-dependent; it relies on vesicular transport (via IFT) for receptor/signalling molecule delivery. Further studies on these ATP-dependent mechanisms may provide insights into potential therapeutic strategies for ciliopathies and other transport-related disorders. L1CAM dysfunction in X-linked L1 syndrome (causing congenital hydrocephalus) may be linked genetically with RAB-3. Griscelli Syndrome (with RAB27A mutation) presented with obstructive hydrocephalus in one case. Carpenter Syndrome: Two of four children with RAB23 mutation had hydrocephalus—no confirmed causal link.
